# The Application of Pulse Flours in the Development of Plant-Based Cheese Analogues: Proximate Composition, Color, and Texture Properties

**DOI:** 10.3390/foods10092208

**Published:** 2021-09-17

**Authors:** Ferawati Ferawati, Mohammed Hefni, Karolina Östbring, Cornelia Witthöft

**Affiliations:** 1 Department of Chemistry and Biomedical Sciences, Linnaeus University, 39231 Kalmar, Sweden; mohammed.hefni@lnu.se (M.H.); cornelia.witthoft@lnu.se (C.W.); 2 Food Industries Department, Faculty of Agriculture, Mansoura University, Mansoura 35516, Egypt; 3 Department of Food Technology Engineering and Nutrition, Lund University, 22362 Lund, Sweden; karolina.ostbring@food.lth.se

**Keywords:** plant-based, cheese analogue, yellow peas, faba beans, texture

## Abstract

Despite the many benefits of pulses, their consumption is still very low in many Western countries. One approach to solving this issue is to develop attractive pulse-based foods, e.g., plant-based cheeses. This study aimed to assess the suitability of different types of pulse flour, from boiled and roasted yellow peas and faba beans, to develop plant-based cheese analogues. Different stabilizer combinations (kappa- and iota-carrageenan, kappa-carrageenan, and xanthan gum) were tested. The results showed that firm and sliceable pulse-based cheese analogues could be prepared using all types of pulse flour using a flour-to-water ratio of 1:4 with the addition of 1% (*w*/*w*) kappa-carrageenan. The hardness levels of the developed pulse-based cheese analogues were higher (1883–2903 g, *p* < 0.01) than the reference Gouda cheese (1636 g) but lower than the commercial vegan cheese analogue (5787 g, *p* < 0.01). Furthermore, the crude protein (4–6% wb) and total dietary fiber (6–8% wb) contents in the developed pulse-based cheese analogues were significantly (*p* < 0.01) higher than in the commercial vegan cheese analogue, whereas the fat contents were lower. In conclusion, flours from boiled and roasted yellow peas and faba beans have been shown to be suitable as raw materials for developing cheese analogues with nutritional benefits.

## 1. Introduction

Pulses are characterized by high contents of protein, dietary fiber, and micronutrients and a low fat content. Increased consumption of pulses has several positive effects with respect to health and the environment [1,2]. The inclusion of pulses in the diet has been reported to reduce the risks of cardiovascular diseases, diabetes, and obesity [2]. In terms of the environmental effects, pulse cultivation results in CO_2_ emissions of only 0.66 kg/kg, which is significantly lower than for livestock production (4–29 kg CO_2_ emission/kg) [1,3]. Despite the many benefits of pulses, their consumption is still low, especially in Western countries, e.g., Sweden (12 g/day) [4].

Pulses can be processed into versatile flour types [5]; however, flours from raw pulses still contain antinutrients, which can negatively affect nutrient absorption, and pulses also have a strong beany flavor. As such, pre-treatment of pulses before milling, such as through boiling and roasting, could be performed to reduce the contents of heat-labile antinutrients and the beany flavor in the pulse flours [6,7]. The pre-treatment could also affect the functional properties of pulse flours to some extent. Flours from boiled and roasted pulses were shown to have high water absorption capacity levels (2–3 g/100 g db) and could form gels at 10–12% concentration, as compared to flour from raw pulses (water absorption capacity of 1 g/100 g and gel forming concentration range of 8–10%) [8]. Flours from boiled and roasted pulses could be utilized in many food products, such as cheese analogues, custards, and bakery goods. The introduction of new pulse-based food products in the market could serve as an approach to increase pulse consumption in Sweden, thereby improving fiber intake, which is currently below the recommended level [4].

Most commercial plant-based cheese analogues on the European market consist of water, coconut oil, starch, stabilizers, salt, vitamins, flavorings, and coloring agents. Consumers are often concerned about the nutritional disadvantages of these oil- and starch-based cheese analogues, whereas the alternatives based on nuts have the issue of being higher in price [9]. Consumers also consider plant-based cheeses as not being as good as their animal-based counterparts in terms of taste and texture [9]; therefore, there is ample room for product development in this area, and the use of pulses to produce plant-based cheese analogues seems promising due to their nutritional value.

Plant proteins differ from milk proteins in terms of their structure and functional properties [10]. Plant proteins have larger molecule sizes and more complex quaternary structures than milk proteins, meaning they cannot form compact gel networks in the way that casein can, which is a crucial step in the production of cheese [10]; therefore, using plant proteins to match the functionality of casein in the development of plant-based cheese will be challenging [11]. Additional ingredients such as stabilizers might help to improve the texture of plant-based cheese analogues [12]. Stabilizers such as carrageenan and xanthan gum are commonly added into cheese analogues (0.3–4% on a mass basis) to improve the firmness of the product and minimize syneresis [12,13,14]. There are three commercial types of carrageenan: kappa, iota, and lambda. Kappa- and iota-carrageenans can form different types of gel in the presence of calcium salts, whereas lambda-carrageenan does not aid in gel formation but increases the viscosity [15]. Xanthan gum is a thickening agent that is commonly combined with gelling agents to increase the elasticity of food gels. Jana et al. [13] suggested that combined stabilizers may synergistically improve the texture of plant-based cheese analogues. The acceptable daily intake (ADI) of carrageenan is 75 mg/kg body weight/day [16], although there is no ADI recommendation for the use of xanthan gum in food [17].

Although there have been several studies on the development of plant-based cheese analogues (mainly from soybean), to the best of our knowledge data are lacking regarding the use of pulse flours to produce plant-based cheese analogues; hence, the objective of this study was to evaluate the suitability of different types of pulse flour (i.e., flours from boiled and roasted yellow peas and faba beans) as raw materials to develop plant-based cheese analogues.

## 2. Materials and Methods

### 2.1. Materials

Yellow pea (var. Clara) and faba bean (var. Gloria) grains were harvested in 2019 and were obtained from Kalmar-Ölands Trädgårdsprodukter, Färjestaden, Sweden. Yellow peas and faba beans were chosen due to their suitability for cultivation in a northern climate and for their abundant supply in Sweden. Rapeseed oil was obtained from a local supermarket in Lund, Sweden. Kappa-carrageenan, iota-carrageenan, and xanthan gum were purchased from Kitchen Lab, Sweden. Nutritional yeast was purchased from Bodystore, Solna, Sweden. Calcium sulphate was purchased from Humlegårdens Ekolager, Sollentuna, Sweden.

### 2.2. Preparation of Pulse Flour

Flour from boiled and roasted yellow peas and faba beans was prepared according to Ferawati et al. [8]. These types of flour were chosen due to their good gelling property and slightly beany flavor [7,8]. Dried yellow peas and faba beans (500 g each) were soaked in tap water at room temperature for 14 h (1:3 *w*/*v*) and were divided into two batches. In the first batch, the beans were boiled in fresh water (1:5 *w*/*v*) for 35 min (yellow peas) and 50 min (faba beans), then dried in a convection oven (50 °C for 16 h) followed by milling (Laboratory mill 120, Perten Instruments AB, Hägersten, Sweden). In the second batch, the beans were roasted at 180 °C for 20 min, cooled to room temperature, then milled as described above. All flour was packed in plastic bags and stored at −20 °C until further experiments.

### 2.3. Preparation of Pulse-Based Cheese Analogues

**Figure 1:**
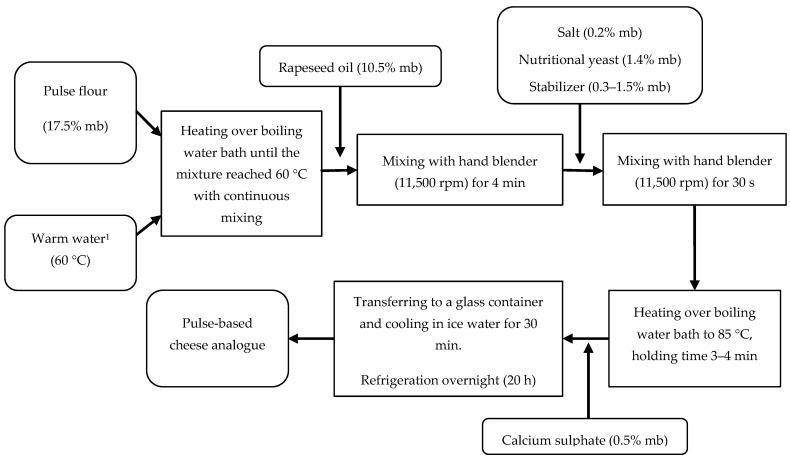
Pulse-based cheese analogue preparation according to Thresher [18]. ^1^ The amount of water was adjusted depending on the stabilizer amount in the formula. Note: mb = mass basis.

Pulse-based cheese analogues were prepared with some modifications from a patent by Thresher (US6777016B2, 2004) [18], as depicted in Figure 1.

#### 2.3.1. Determination of Pulse-Flour-to-Water Ratio

Five different ratios of flour from boiled peas (BYP) to water (1:3, 1:3.5, 1:4, 1:4.5, 1:5 *w*/*w*) were examined in the formulation, aiming to produce a firm and sliceable cheese analogue sample. The ratio of 1:4 was used as a starting point to determine the effects of the different water addition levels on the texture of the cheese analogues [18]. A low concentration of kappa-carrageenan (0.3%) was needed in the formula to achieve a sample that could be sliced. All pulse-based cheese analogues were prepared as outlined in Figure 1 in duplicate and were subjected to texture analysis in triplicate (Section 2.4.3). The samples were also visually evaluated to provide an additional description based on the appearance of the produced cheese analogues. The formula with the best flour-to-water ratio out of those investigated in this study was used to evaluate the effects of the type and concentration of stabilizers.

#### 2.3.2. Determination of Stabilizer Type and Concentration

Kappa-carrageenan (KCG) was evaluated at four different concentrations (0.3%, 0.5%, 1%, and 1.5%, *w*/*w*). Additionally, combinations of kappa-carrageenan with iota-carrageenan (ICG) or xanthan gum (XG) were explored at different ratios (10:90, 50:50, 90:10) with a total stabilizer concentration of 1% (*w*/*w*) in the formula. All samples were subjected to texture analysis in triplicate, as described in Section 2.4.3. The samples were also visually evaluated to provide an additional description based on the appearance of the produced cheese analogues.

#### 2.3.3. Preparation of Cheese Analogues Using Different Types of Pulse Flour

Pulse-based cheese analogues were produced in duplicate trials using flours made from boiled yellow peas (BYP), roasted yellow peas (RYP), boiled faba beans (BFB), and roasted faba beans (RFB) according to the findings derived from preliminary trials (Section 2.3.1 and Section 2.3.2, Table 1). Two commercial products, a dairy Gouda cheese (AxFood Sverige AB, Stockholm, Sweden) and a vegan cheese analogue made from coconut oil and starch (Violife Foods, Thessaloniki, Greece), were used as references. The references and the pulse-based cheese analogues were subjected to analysis in terms of the proximate composition, color, and texture.

### 2.4. Characterization of Pulse-Based Cheese Analogues

#### 2.4.1. Proximate Composition

The crude protein content was determined according to the Dumas combustion method AOAC 990.03 using a protein analyzer (Flash EA 1112 Series, Thermo Scientific, Waltham, MA, USA) [19]. A conversion factor of 6.25 was used to calculate the protein content. The fat content was determined by extraction with petroleum ether (Soxtec Avanti, Tecator AB, Höganäs, Sweden) according to AOAC 920.39 [20]. The carbohydrate content was calculated as the difference between 100 and the sum of the moisture, ash, protein, fat, and total dietary fiber content. The total dietary fiber (TDF) content was analyzed according to AOAC 991.43 using the K-TDFR kit (Megazyme, Wicklow, Ireland) [21]. For ash content determination (AOAC 923.03), samples were transferred into porcelain crucibles and incinerated in a furnace at 550 °C for 16 h [22]. The moisture content was determined by drying the sample in an oven at 105 °C for 16 h [23]. All analyses were performed in triplicate.

#### 2.4.2. Color Properties

The surface color of the cheese analogues was measured using a colorimeter (CM-700d, Konica Minolta Sensing, Inc, Tokyo, Japan) and the L*, a*, and b* values of the samples were also recorded. The L* value represents the lightness of a sample from black to white. The a* (−green to +red) and b* (−blue to +yellow) values represent the chromatic aspects of a sample [24]. The analyses were performed in triplicate.

#### 2.4.3. Texture Properties

Texture profile analysis (TPA) was conducted using a texture analyzer (TVT-300XP, Perten Instruments AB, Hägersten, Sweden). The measurements were performed according to Le Tohic et al. [25] with some modifications. Samples of cheese analogues were cut into 20 mm cubes immediately after removal from the fridge (4 °C). Two compression–decompression cycles were carried out using a cylindrical probe (diameter of 18 mm), separated by a time interval of 5 s at a rate of 1 mm/s. The probe compressed the sample by 5 mm from the initial height (20 mm). The analysis was performed in triplicate.

### 2.5. Statistical Analysis

Data on the textural properties of the samples obtained from each trial and the proximate composition, color, and texture of the developed pulse-based cheese analogues were expressed as means (*n* = 6, triplicate analysis from two independent trials) ± standard deviation (sd). The data on the proximate composition were presented on a wet basis (wb). One-way ANOVA and post-hoc Tukey’s test were used to determine significant differences between samples in each trial and differences between cheese analogues prepared from different types of pulse flour and references. The significance level was set at <0.05. Statistical analyses were performed using Graphpad Prism software version 9.1.1–2021.

## 3. Results and Discussion

### 3.1. Evaluation of Processing Conditions

#### 3.1.1. Effects of Pulse-Flour-to-Water Ratio on Texture

The amount of water significantly affected the texture of the pulse-based cheese analogue samples (*p* < 0.05) (Table 2). The sample with the lowest amount of water (a flour-to-water ratio of 1:3) had the highest levels of hardness, chewiness, and springiness as compared to the other ratios, although it had a gritty texture and a small amount of oil was observed leaking out from the product matrix (Table 2). The sample with the highest amount of water (a flour-to-water ratio of 1:5) had a smooth appearance but had the lowest levels of hardness, chewiness, and springiness amongst the samples. These results were expected because incorporation of increasing amounts of water into a gel network is reflected by a softer and smoother texture [26]. The flour-to-water ratio of 1:4 resulted in a firm and sliceable sample, which was also the easiest to handle during preparation, confirming findings by others [18]; thus, the flour-to-water ratio of 1:4 was used in subsequent trials.

#### 3.1.2. Effect of Stabilizers on Texture

Increasing the amount of kappa-carrageenan (KCG) from 0.3% to 1.5% in the formula led to significant (*p* < 0.05) increases in the hardness, chewiness, and cohesiveness of the pulse-based cheese analogue (Table 2). Similarly, Hánákova et al. [27] observed that the use of 1% (*w*/*w*) KCG in a partial dairy cheese analogue formulation resulted in a significant increase in hardness compared with a control sample without the addition of KCG. This was due to the ability of KCG to form a three-dimensional gel structure and KCG’s interaction with other components in the matrix, enhancing the firmness of the cheese analogue [27]. The combination of stabilizers did not improve the cohesiveness of the cheese analogues. This was shown by the lower hardness, chewiness, and cohesiveness values in the samples made with combined stabilizers than the sample using KCG only (Table 3). This is in contrast to the findings of Jana et al. [13], who suggested that a combination of stabilizers might exhibit a synergistic effect by altering the gelling characteristics [13]. These conflicting results might be due to the different types of raw material and stabilizers used.

The final pulse flour-to-water ratio of 1:4 with 1% (*w*/*w*) KCG resulted in a pulse-based cheese analogue with a hardness similar to the dairy Gouda cheese but lower than the commercial vegan cheese analogue (Table 2). The use of kappa-carrageenan (KCG) at 1% in the formula is still within the acceptable daily intake range (75 mg/kg body weight/day~5.3 g/70 kg body weight/day) [16]; therefore, these moisture and stabilizer content levels were chosen to be used in the proceeding evaluation of how different types of pulse flour affect texture.

### 3.2. Characterization of Plant-Based Cheese Analogues from Different Types of Pulse Flour

#### 3.2.1. Proximate Composition

The pulse-based cheese analogues had a protein content between 4–7 g/100 g wb, which was higher than the commercial vegan cheese analogue but lower than the Gouda cheese (Table 4). A previous study reported a higher protein content (20 g/100 g wb) in a cheese analogue made from soy milk [28], possibly caused by the different production methods used. Both, the Gouda cheese and soybean cheese analogue were produced with a traditional curdling method that concentrated the protein [28,29]. The fat content in the pulse-based cheese analogues was significantly lower (10 g/100 g wb) than in the commercial reference samples (24 and 28 g/100 g wb) (Table 4) but higher than reported data from a soymilk cheese analogue (7 g/100 g wb) [28]. In the present study, rapeseed oil was used as the fat source, whereas milk fat and coconut oil were used in the reference samples of Gouda and vegan cheese analogue, respectively.

The carbohydrate contents in the pulse-based cheese analogues, ranging from 3–8 g/100 g wb, were higher than in the reference Gouda cheese (0.6 g/100 g wb) but far below the content in the vegan cheese analogue (25 g/100 g wb) (Table 4). The high carbohydrate content in the vegan cheese analogue might be caused by the high level of starch in the composition. The pulse-based cheese analogues had 3–4 times higher contents of dietary fiber (7–8 g/100 g wb) than the Gouda cheese, whereas the fiber contents of both the vegan cheese analogue (Table 4) and the soymilk cheese analogue [28] were below 1 g/100 g wb. The fiber content in the pulse-based cheese analogues was derived mainly from the husks. The pulse based cheese analogues could be categorized as high-fiber products according to European regulation (at least 6 g fiber/100 g product) [30]. The ash content in the pulse-based cheese analogues was lower than the content in the references (Table 4). Overall, these results demonstrate that pulse-based cheese analogues could be considered functional foods.

#### 3.2.2. Color Properties

**Figure 2:**
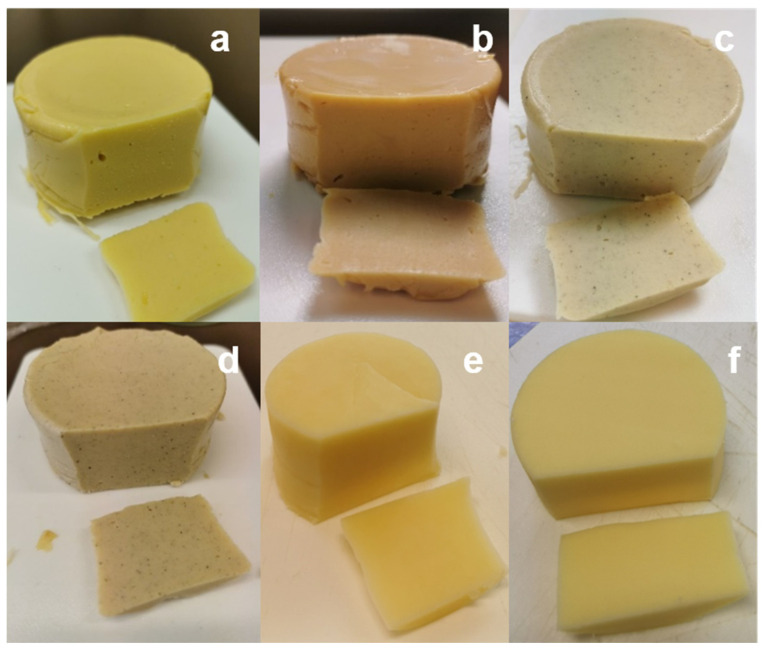
Pulse-based cheese analogues: BYP (**a**); RYP (**b**); BFB (**c**); RFB (**d**); reference samples Gouda cheese (**e**); vegan cheese analogue (**f**). BYP = boiled yellow pea flour; RYP = roasted yellow pea flour; BFB = boiled faba bean flour; RFB = roasted faba bean flour.

The color assessment of the pulse-based cheese analogues showed distinct differences between the samples. Most pulse-based cheese analogues had lower lightness (L*) and yellowness (b*) levels compared with the reference samples (Table 5). The pulse-based cheese analogue made from boiled yellow pea flour (BYP) had the closest resemblance in color to the commercial Gouda cheese (Table 5; Figure 2). The cheese analogues prepared from faba bean flour (BFB and RFB) had noticeable dark spots caused by the seed husks (Figure 2). In general, cheese analogues made from roasted pulse flours tended to have higher redness (increased a* value) but lower lightness (decreased L* value) and yellowness (decreased b* value) levels than the cheese analogues made from boiled pulse flours. This difference might be due to Maillard reactions that occurred during the preparation of the flour from roasted pulses leading to a darker color [31].

#### 3.2.3. Texture Properties

The cheese analogues made from faba bean flour (BFB and RFB) had higher levels (*p* < 0.01) of hardness and chewiness than the samples made from yellow pea flour (Table 5). This may have been due to the different contents of protein, starch, and fiber in the faba bean and yellow pea flours. Joshi et al. [32] observed that the starch-to-protein ratio significantly affected the strength of a lentil protein gel (15% total solids *w*/*v*); however, the composition of the starch also affected the gel strength, while amylose impaired the gel strength [32]. This may have been due to a lesser tendency of amylose to interact with protein and preferred amylose–amylose interactions in the composite gel system [32]. For yellow peas, an amylose content of around 50% was reported, while for faba beans a lower content range of around 34–40% was reported [33,34]. This might explain why the cheese analogues made from yellow pea flour showed levels of lower hardness even though yellow peas contain a higher proportion of carbohydrate (Table 4). Moreover, the content of dietary fiber was found to improve the gel strength due to the formation of a stable network structure resulting from stabilizing the water phase and the filling effect [35].

The different treatment used to produce pulse flours also had a significant effect on the texture of the cheese analogues (Table 5). The samples made of flour from roasted pulses (RYP and RFB) were harder than the sample prepared using flour from boiled pulses (Table 5). Variations in the degree of starch gelatinization and protein denaturation due to different treatments on pulses prior to milling were assumed to be responsible for the differences in texture properties. Boiling provides the moisture and heat required for starch gelatinization and protein denaturation, whereas roasting uses hot air circulation, which might not be sufficient to support complete gelatinization and protein denaturation [36,37]; therefore, flour from roasted pulses is assumed to contain less gelatinized starch and denatured protein than flour made from boiled pulses [37]. During the heating step in the production of cheese analogues, the ungelatinized starch and undenatured protein fractions in the roasted pulse flour continued to gelatinize, denature, and interact with each other, thereby forming a network; thus, this resulted in a harder gel texture in the samples made of flour from roasted pulses than the samples made of flour from boiled pulses. There is no literature available regarding the texture properties of cheese analogues made from pulse flour.

All pulse-based cheese analogues were harder, chewier, and less cohesive than the reference Gouda cheese (Table 5). This may have been due to higher contents (three- to eight-fold higher) of carbohydrate (starch) [38] and the use of kappa-carrageenan in the pulse-based cheese analogues [12]. The reference commercial vegan cheese analogue had the greatest hardness and chewiness amongst the samples (Table 5), which may have been due to the starch content of 25 g/100 g wb immobilizing the water in the matrix [38]. The springiness levels of all pulse-based cheese analogues were similar to the vegan cheese analogue but higher than the Gouda cheese reference (Table 5).

This study is subject to certain limitations that could be addressed in future research. Firstly, during determination of the processing conditions, only one type of pulse flour was used (flour from boiled yellow pea) in order to limit the numbers of variables during the trial. Thereby, effects on the texture of the cheese analogues due to different chemical compositions between pulse species might be overlooked. Secondly, the trials were performed in a sequence, which may have discounted the other flour-to-water ratios and the different concentrations of kappa-carrageenan used to produce cheese analogues with similar texture properties as the reference Gouda cheese.

## 4. Conclusions

Flours from boiled and roasted yellow peas and faba beans were deemed to be suitable for use as raw materials to produce plant-based cheese analogues. The pulse-based cheese analogues can be viewed as functional foods due to their high fiber contents (6–8 g/100 g product). The developed pulse-based cheese analogues could potentially be healthier alternatives in the current plant-based cheese market and could serve as vehicles to increase pulse consumption. A sensory study to assess the acceptability of the products should be conducted to complement the present results. An optimization study to develop the flavor profiles of pulse-based cheese analogues by adding microbial starter cultures could also be explored.

## Figures and Tables

**Table 1:** The recipe for the preparation of pulse-based cheese analogues using different types of pulse flour.

Ingredient	Amount (% in Mass)
Water	68.9
Pulse flour	17.5
Rapeseed oil	10.5
Nutritional yeast	1.4
Kappa-carrageenan	1.0
Calcium sulphate	0.5
Salt	0.2

**Table 2:** The texture properties of pulse-based cheese analogues from yellow pea flour, showing the effects of the water ratio and stabilizer concentration ^1^.

Sample	Springiness	Chewiness (g)	Hardness (g)	Cohesiveness	Description
Pulse flour-to-water ratio					
1:3	1.21 ± 0.20 ^a^	456 ± 77 ^a^	984 ± 82 ^a^	0.30 ± 0.04 ^b^	Very firm, oil leaked out of the matrix. Gritty texture.
1:3.5	1.09 ± 0.09 ^a^	328 ± 14 ^b^	698 ± 17 ^b^	0.32 ± 0.02 ^ab^	Sliceable, very firm, compact, gritty texture.
1:4	1.12 ± 0.15 ^a^	271 ± 22 ^bc^	581 ± 42 ^c^	0.32 ± 0.04 ^ab^	Sliceable, firm, compact.
1:4.5	1.03 ± 0.04 ^a^	212 ± 15 ^cd^	446 ± 24 ^d^	0.36 ± 0.02 ^a^	Sliceable, smooth appearance, soft when touched.
1:5	1.06 ± 0.04 ^a^	186 ± 17 ^d^	389 ± 12 ^e^	0.36 ± 0.02 ^a^	Sliceable, smooth appearance, tofu-like texture.
Stabilizer concentration					
Control	0.85 ± 0.06 ^c^	65 ± 3.3 ^e^	99 ± 9.9 ^e^	0.63 ± 0.03 ^a^	Very soft. Sticky when sliced.
KCG 0.3%	1.07 ± 0.08 ^a^	261 ± 15 ^d^	536 ± 24 ^d^	0.37 ± 0.04 ^d^	Sliceable. Soft tofu-like texture.
KCG 0.5%	1.01 ± 0.11 ^ab^	435 ± 34 ^c^	851 ± 19 ^c^	0.45 ± 0.06 ^c^	Sliceable. Soft tofu-like texture.
KCG 1%	0.94 ± 0.05 ^bc^	990 ± 57 ^b^	1754 ± 37 ^b^	0.54 ± 0.05 ^b^	Sliceable. Very firm. Some tiny air bubbles.
KCG 1.5%	0.91 ± 0.06 ^c^	1415 ± 103 ^a^	2297 ± 105 ^a^	0.61 ± 0.02 ^a^	Sliceable. Very firm. A tiny amount of oil leaked out of the matrix. Some big air bubbles (diameter~2 mm) with entrapped oil.
References					
Gouda cheese	0.86 ± 0.01	1253 ± 87	1636 ± 108	0.76 ± 0.01	
Vegan cheese analogue	0.95 ± 0.05	3906 ± 283	5787 ± 121	0.67 ± 0.03	

^1^ All data are presented as means ± sd (triplicate analyses from two independent trials, *n* = 6). For each trial, means within a column with different letters are significantly different (Tukey’s test, *p* < 0.05). KCG = kappa-carrageenan.

**Table 3:** The texture properties of cheese analogues made with different combinations of stabilizers ^1^.

Sample	Springiness	Chewiness (g)	Hardness (g)	Cohesiveness
KCG—ICG 90:10	0.98 ± 0.04 ^c^	940 ± 12 ^c^	1682 ± 11 ^b^	0.54 ± 0.02 ^c^
KCG—ICG 50:50	1.06 ± 0.03 ^b^	531 ± 2 ^d^	1058 ± 82 ^c^	0.41 ± 0.02 ^de^
KCG—ICG 10:90	1.35 ± 0.10 ^a^	255 ± 25 ^d^	479 ± 6 ^f^	0.34 ± 0.02 ^e^
KCG—XN 90:10	0.97 ± 0.02 ^bc^	810 ± 53 ^cd^	1593 ± 19 ^b^	0.45 ± 0.05 ^cd^
KCG—XN 70:30	1.03 ± 0.03 ^b^	490 ± 47 ^d^	991 ± 27 ^d^	0.41 ± 0.03 ^de^
KCG—XN 50:50	0.95 ± 0.05 ^bc^	328 ± 7 ^d^	718 ± 47 ^e^	0.36 ± 0.04 ^e^
KCG 100	0.97 ± 0.03 ^bc^	947 ± 3 ^c^	1736 ± 32 ^b^	0.50 ± 0.02 ^c^
Gouda cheese	0.86 ± 0.01 ^c^	1253 ± 87 ^b^	1636 ± 108 ^b^	0.76 ± 0.01 ^a^
Vegan cheese analogue	0.95 ± 0.06 ^bc^	3906 ± 283 ^a^	5787 ± 121 ^a^	0.67 ± 0.04 ^b^

^1^ All values are presented as means ± sd (triplicate analyses, *n* = 3). For each parameter, means within a row with different letters are significantly different (Tukey’s test, *p* < 0.05). KCG = kappa-carrageenan; ICG = iota-carrageenan; XN = xanthan gum.

**Table 4:** The proximate composition ^1^ of pulse-based cheese analogues and reference products.

Parameter	BYP	RYP	BFB	RFB	Gouda Cheese	Vegan Cheese Analogue
Protein	4.2 ± 0.0 ^e^	4.4 ± 0.0 ^d^	6.2 ± 0.0 ^c^	6.6 ± 0.0 ^b^	23.5 ± 0.2 ^a^	0.0 ± 0.0 ^f^
Fat	10.6 ± 0.4 ^c^	10.4 ± 0.3 ^c^	10.8 ± 0.1 ^c^	9.9 ± 0.1 ^d^	27.6 ± 0.2 ^a^	19.2 ± 0.0 ^b^
Carbohydrate	7.2 ± 0.5 ^c^	8.3 ± 0.2 ^b^	3.3 ± 0.6 ^e^	5.3 ± 0.4 ^d^	0.6 ± 0.1 ^f^	25.2 ± 0.1 ^a^
Total dietary fiber	7.1 ± 0.3 ^b^	6.5 ± 0.3 ^c^	7.9 ± 0.5 ^a^	7.7 ± 0.5 ^ab^	2.3 ± 0.4 ^d^	0.2 ± 0.0 ^e^
Ash	1.3 ± 0.1 ^d^	1.3 ± 0.0 ^d^	1.4 ± 0.0 ^cd^	1.5 ± 0.0 ^c^	3.1 ± 0.1 ^a^	2.2 ± 0.1 ^b^
Moisture	69.5 ± 0.1 ^b^	68.9 ± 0.3 ^c^	70.3 ± 0.1 ^a^	68.9 ± 0.1 ^c^	42.9 ± 0.8 ^e^	52.8 ± 0.2 ^d^

^1^ All data are in g/100 g wb. All values are presented as means ± sd (triplicate analyses from two independent trials, *n* = 6). For each parameter, means within a row with different letters are significantly different (Tukey’s test, *p* < 0.05). BYP = boiled yellow pea flour; RYP = roasted yellow pea flour; BFB = boiled faba bean flour; RFB = roasted faba bean flour.

**Table 5:** Color and texture properties ^1^ of pulse-based cheese analogues made from different types of pulse flour and the reference products.

Parameter	BYP	RYP	BFB	RFB	Gouda Cheese	Vegan Cheese Analogue
Color properties						
L*	75 ± 0.9 ^c^	69 ± 0.2 ^f^	74 ± 0.9 ^d^	72 ± 0.4 ^e^	78 ± 0.6 ^b^	84 ± 0.1 ^a^
a*	3 ± 0.2 ^c^	6 ± 0.1 ^a^	1 ± 0.1 ^e^	2 ± 0.1 ^d^	3 ± 0.1 ^c^	4 ± 0.2 ^b^
b*	27 ± 0.8 ^b^	23 ± 0.7 ^d^	17 ± 0.4 ^e^	17 ± 0.4 ^e^	25 ± 0.6 ^c^	32 ± 1.1 ^a^
Texture properties						
Springiness	0.94 ± 0.05 ^a^	0.94 ± 0.02 ^a^	0.92 ± 0.02 ^ab^	0.94 ± 0.03 ^a^	0.86 ± 0.01 ^b^	0.95 ± 0.06 ^a^
Chewiness (g)	1089 ± 20 ^d^	1706 ± 188 ^c^	1247 ± 52 ^d^	2024 ± 42 ^b^	1253 ± 87 ^d^	3906 ± 283 ^a^
Hardness (g)	1883 ± 12 ^e^	2537 ± 209 ^c^	2121 ± 95 ^d^	2903 ± 93 ^b^	1636 ± 108 ^f^	5787 ± 121 ^a^
Cohesiveness	0.56 ± 0.02 ^c^	0.67 ± 0.02 ^b^	0.58 ± 0.03 ^c^	0.70 ± 0.01 ^b^	0.76 ± 0.01 ^a^	0.67 ± 0.1 ^b^

^1^ All values are presented as means ± sd (triplicate analyses from two independent trials, *n* = 6). For each parameter, means within a row with different letters are significantly different (Tukey’s test, *p* < 0.05). BYP = boiled yellow pea flour; RYP = roasted yellow pea flour; BFB = boiled faba bean flour; RFB = roasted faba bean flour.

## Data Availability

All data generated or analyzed during this study are included in this article.
